# Availability, price and affordability of anticancer medicines in Jiangsu Province, China: a cross-sectional survey study

**DOI:** 10.3389/fpubh.2025.1729325

**Published:** 2025-12-18

**Authors:** Yanping Yao, Yulu Zhu, Zhuying Jing, Lihong Gao, Haomin Zhu, Jia Wang, Shanhui Wang, Zhaoliu Cao, Tiantian Tao, Xin Li

**Affiliations:** 1 Department of Pharmacy, Suzhou Xiangcheng People's Hospital, Suzhou, Jiangsu, China; 2 Department of Pharmaceutical Regulatory Science and Pharmacoeconomics, School of Pharmacy, Nanjing Medical University, Nanjing, China; 3 Department of Health Policy, School of Health Policy and Management, Nanjing Medical University, Nanjing, China; 4 Jiangning District Center for Disease Control and Prevention, Nanjing, Jiangsu, China; 5 Nanjing City Qixia District Hospital, Nanjing, Jiangsu, China; 6 Department of Pharmacy, The Second People’s Hospital of Changzhou, The Third Affiliated Hospital of Nanjing Medical University, Changzhou, Jiangsu, China; 7 Center for Global Health, School of Public Health, Nanjing Medical University, Nanjing, China

**Keywords:** affordability, anticancer medicines, availability, China, price, WHO/HAI

## Abstract

**Background:**

To evaluate anticancer medicines’ availability, price and affordability in nine prefecture-level cities, Jiangsu Province.

**Methods:**

Based on the standard methods recommended by the World Health Organization (WHO) and the Health Action International (HAI), a cross-sectional survey was performed to collect information about 24 essential anticancer medicines (EAMs) and 17 innovative anticancer medicines (IAMs) in hospitals and 45 community pharmacies in Jiangsu Province from July to November 2021.

**Results:**

In general, the availability of EAMs during the study period was higher than that of IAMs, and the availability of the Lowest-priced generics (LPGs) was higher than that of the originator brands (OBs). Anticancer medicines in hospitals were more available than those in community pharmacies. Specifically, the availability of anticancer medicines positively correlated with different cities’ economic status. However, there were no significant changes in the availability of IAMs among different cities. The median price ratio (MPR) value of OBs was greater than that of LPGs, and both were greater than one. There were no significant changes in the MPR value of EAMs among different economic regions (*p* > 0.05). The price of IAMs was significantly greater than the median price of 18 European countries. There were significant changes in the affordability of EAMs and IAMs among different cities (*p* < 0.05). In this research context, IAMs had poor affordability, but LPGs had good affordability for the residents. The affordability of urban residents was better than that of rural residents.

**Conclusion:**

The availability of anticancer medicines in community pharmacies was low, especially in intravenous anticancer preparations. The Chinese government formulated a dual-channel policy to improve the availability of anticancer medicines in community pharmacies. However, implementing the dual-channel policy required the effective interaction and cooperation of the hospitals, community pharmacies, medical insurance, and other departments. The price of IAMs was still the main obstacle to receiving treatment for cancer patients; therefore, the government should further optimize the price of IAMs through various strategies to achieve a reasonable price. Expanding the coverage of EAMs was conducive to improving treatment affordability for cancer patients.

## 1 Introduction

Cancer remains the leading cause of death globally and a leading barrier to improving life expectancy. According to the data released by the International Agency for Research on Cancer (IARC) in 2021, there were 19.3 million new cancer cases and 10 million deaths worldwide in 2020, with approximately 4.57 million new cases in China alone. IARC further predicts that global cancer incidence will rise to 28.4 million new cases by 2040, with the most significant increase in cases expected in the low-and middle-income countries (1).

Tumor diseases have garnered global attention not only because of their high mortality rates but also due to their significant financial burden on patients. The availability and affordability of anticancer medicines are critical to successfully implementing prevention, screening and treatment programs, particularly in developing countries. However, factors such as the high price of anticancer medicines, imperfect supply systems and suboptimal treatment quality limit patients’ access to these life-saving therapies (2). According to the WHO Global Action Plan, the availability of anticancer medicines is set to reach 50% by 2025 (3). However, data from international sources reveal significant gaps in achieving this goal. A Mexican study showed that the mean availability of anticancer medicines in public hospitals and private pharmacies was 61.2 and 67.5%, respectively (4). Public hospitals achieve only 43% availability of essential anticancer drugs in Pakistan (5). The situation appears particularly concerning in China. Hubei Province reports availability rates of just 13.7 and 6.67% for originator brands (OBs) in tertiary and secondary hospitals, respectively, with lower-priced generics (LPGs) performing better at 62.83 and 42.92% (6). Our previous Study in Jiangsu Province (2012–2016) documented a worrying decline in availability for both OBs (7.79–5.71%) and LPGs (36.29–32.67%) among 40 essential anticancer medicines (7). Financial barriers compound these accessibility challenges. High out-of-pocket healthcare expenditures remain a systemic challenge, with unaffordable medication prices, particularly for innovative anticancer medicines (IAMs), representing the primary reason for treatment discontinuation. In 2016, China implemented its “National Drug Price Negotiation Policy” targeting patented and exclusively produced high-cost innovative therapeutics (including anticancer targeted agents and orphan drugs). Led by the National Healthcare Security Administration (NHSA), multidisciplinary negotiation teams comprising clinical specialists, pharmaceutical scientists, and health economists engaged in direct price negotiations with pharmaceutical manufacturers. These negotiations employed volume-based procurement strategies, leveraging therapeutic clinical value, international reference pricing, and patient population needs to secure price concessions. Successful negotiations resulted in inclusion in the National Reimbursement Drug List (NRDL), with select agents additionally qualifying for the National Essential Medicines List (NEML). Despite achieving an average 61.71% price reduction across 67 negotiated agents in 2021, numerous anticancer therapies remained unavailability in hospitals due to performance metrics, particularly drug expenditure-to-sales ratio restrictions and inventory management protocols. These policies restricted the procurement of National Negotiated Drugs. Therefore, China formally instituted the “National Negotiated Drug Dual-Channel Management Policy” in May 2021. This policy established a parallel distribution system including hospital and community pharmacy to ensure availability of anticancer medicines that remain excluded from hospital formularies. NHSA selected “designated retail community pharmacies” meeting stringent criteria—including cold-chain storage capacity and on-site pharmacist verification—as official supply points for negotiated drugs. Through this “dual-channel” framework, patients purchased negotiated drugs through these dual-channel community pharmacies receive identical medical insurance reimbursement policies as those obtaining the same medications via hospitals. Additionally, the NHSA regularly update the dual-channel drug directory and oversee the drug supply and service quality of these dual-channel community pharmacies.

There were several limitations in the existing literature on related researches. Only a limited number of anticancer medicines were evaluated independently of the values of accessibility and affordability. The accessibility of anticancer medicines in Eastern China, where the incidence of cancer diseases is highest among different regions, has remained unclear since 2016. The comparison between the accessibility and affordability of essential anticancer medicines (EAMs) and IAMs warrants further studies. The evaluation of the accessibility of anticancer medicines in community pharmacies has not yet been investigated in China. Most crucially, existing studies had not yet evaluated the impact of National Drug Price Negotiation policy and the Dual-channel policy on the availability, price, and affordability of anticancer medicines. We conducted a comprehensive survey using standardized WHO methodology to address these gaps.

In this study, we adopted the cross-sectional survey method, and the survey time was from July 2021 to November 2021. Based on the WHO/HAI methodological recommendations, we assessed the accessibility, pricing, and affordability of 24 EAMs and 17 IAMs across 45 hospitals and 45 community pharmacies in 9 prefecture-level cities throughout Jiangsu Province. The relevant information covers drug prices, specifications, manufacturers, dosage forms, availability, etc.

## 2 Materials and methods

### 2.1 Research design

We selected nine prefecture-level cities in Jiangsu Province according to the GDP level of each city in 2020 (8). These consisted of four high-income cities: Nanjing, Wuxi, Changzhou, Suzhou; three middle-income cities: Yangzhou, Yancheng, Xuzhou; and two low-income cities: Huai’an, Lianyungang. The hospitals were selected according to the following criteria: (I) Inclusion of the highest-level tertiary general hospitals in each region. (II) Requirement of official certification for oncology diagnosis and treatment. (III) Random selection of 2–3 secondary or tertiary hospitals within a three-hour drive from urban centers. The final sample included 45 hospitals: 27 tertiary hospitals (including 6 specialized oncology hospitals) and 18 secondary hospitals. Community pharmacies were identified from Jiangsu Medical Insurance Bureau’s 2021 dual-channel pharmacy list (9). In regions with fewer than five listed dual-channel pharmacies or where no official list was available, the community pharmacies near the investigated hospitals or Institution-affiliated pharmacies were selected instead. This selection process yielded 45 community pharmacies and all of them were dual-channel pharmacies.

### 2.2 Selection of anticancer medicines

Based on recommendations from oncologists and local health statistics, we focused on five malignancies with high morbidity and mortality in our study: lung cancer, gastric cancer, esophageal cancer, liver cancer, and colorectal cancer. EAMs were selected according to the following criteria: (I) Inclusion in China’s National Essential Medicines List (NEML). (II) Listing on the WHO Essential Medicines List (EML). (III) Approved for market in China before 2016. Following this selection process, 24 EAMs were selected and relevant information was provided in Table 1.

**Table 1:** Basic information of essential anticancer medicines.

Drug name	Dosage form	Strength	DDD (mg)	Main indications	International reference price	Chinese NEML	WHO EML	Medical insurance catalog	OBs	LPGs
Cyclophosphamide	inj	200 mg	92	Leukemia	2.08	Yes	Yes	Yes	Yes	Yes
Ifosfamide	inj	1 g	140	OC	26.73	Yes	Yes	Yes	Yes	Yes
Methotrexate	tab/cap	2.5 mg	2	Leukemia	0.063	Yes	Yes	Yes	No	Yes
Mercaptopurine	tab/cap	50 mg	147	Leukemia	2.24	Yes	Yes	Yes	No	Yes
Cytarabine	inj	100 mg	41	Leukemia	3.48	Yes	Yes	Yes	Yes	Yes
Hydroxyurea	tab/cap	500 mg	800	HNC	0.22	Yes	Yes	Yes	No	Yes
Fluorouracil	inj	250 mg	1,050	BC	2.60	Yes	Yes	Yes	No	Yes
Gemcitabine	inj	200 mg	182	NSCLC	6.28	Yes	Yes	Yes	Yes	Yes
Etoposide	inj	20 mg	68	NSCLC	0.40	Yes	Yes	Yes	No	Yes
Daunorubicin	inj	20 mg	61	Leukemia	19.32	Yes	Yes	Yes	No	Yes
Vincristine	inj	1 mg	0.29	Leukemia	2.54	Yes	Yes	Yes	No	Yes
Paclitaxel	inj	100 mg	14	OC	11.08	Yes	Yes	Yes	Yes	Yes
Cisplatin	inj	10 mg	5	OC	2.75	Yes	Yes	Yes	No	Yes
Oxaliplatin	inj	50 mg	11	CRC	28.88	Yes	Yes	Yes	Yes	Yes
Carboplatin	inj	150 mg	24	OC	16.01	Yes	Yes	Yes	Yes	Yes
Arsenic trioxide	inj	5 mg	10	Leukemia	--	Yes	Yes	Yes	No	Yes
Asparaginase	inj	10,000 IU	1700 IU	Leukemia	52.88	Yes	Yes	Yes	No	Yes
Calcium folinate	inj	0.1 g	425	Auxiliary	--	Yes	Yes	Yes	No	Yes
Capecitabine	tab/cap	500 mg	2,833	CRC	1.67	Yes	Yes	Yes	Yes	Yes
Tamoxifen	tab/cap	10 mg	20	BC	0.08	Yes	Yes	Yes	Yes	Yes
Sodium mesylate	inj	400 mg	1,680	Auxiliary	3.01	Yes	Yes	Yes	Yes	Yes
Imatinib	tab/cap	100 mg	400	Leukemia	0.69	Yes	Yes	Yes	Yes	Yes
Rituximab	inj	100 mg	116	LC	136.72	Yes	Yes	Yes	Yes	Yes
Trastuzumab	inj	440 g	20	BC	–	Yes	Yes	Yes	Yes	Yes

DDD, defined daily dose; NEML, National Essential Medicines Lists; WHO/EML, World Health Organization/Essential Medicines Lists; LPGs, lowest-price generics; OBs, originator brands; inj, injection; TAB, tablet; CAP, capsule; YES, on the list; NO, not on the list; OC, ovarian cancer; HNC, head and neck cancer; CRC, colorectal cancer; BC, breast cancer; LC, lymphoid carcinoma; NSCLC, non-small cell lung cancer.

Meanwhile, the selection of Innovative Anticancer Medicines (IAMs) was based on the definition and classification outlined in the Guiding Principles of Clinical Application of Innovative Anticancer Medicines (GPCAIAMs 2020) issued by the National Health Commission (NHC). The inclusion criteria were: (I) based on the classification method by GPCAIAMs 2020. (II) Included in the GPCAIAMs 2020 formulary. (III) If an IAM was also listed as an essential medicine, it was preferentially evaluated as an EAM. (IV) Approved in China before 2016. This process identified 17 clinically representative IAMs and divided them into three categories: monoclonal antibodies (*n* = 2), Targeted drugs (*n* = 11), and other targeted drugs (*n* = 4). The relevant information on IAMs is summarized in Table 2. Due to limited data on IAM generics, only the originator drugs were evaluated for availability and pricing. Consequently, procurement bid (OB) and local procurement guideline (LPG) data were collected for EAMs, while only OB information was obtained for IAMs. Finally, the Study evaluated a total of 41 anticancer agents, categorized by therapeutic indication as follows: leukemia (*n* = 11), lung cancer (*n* = 7), breast cancer and colorectal cancer (4 agents each), ovarian cancer (*n* = 3), other systemic malignancies (*n* = 10), and supportive care medications (*n* = 2).

**Table 2:** Basic information of innovative anticancer medicines.

Drug name	mPFS (month)	Dosage form	Strength	DDD (mg)	NEML	WHO EML	Medical insurance medicine	Main indication	Registration category
Bevacizumab	11.20 (27)	inj	100 mg	50	No	Yes	Yes	Colorectal Cancer	Biological Products 3.1
Nimotuzumab	7.29 (28)	inj	50 mg	14	No	No	Yes	Nasopharyngeal Carcinoma	Biological Products 1
Dasatinib	4 (29)	tab/cap	50 mg	100	No	Yes	Yes	Leukemia	Chemical Drugs 5.1
Gefitinib	10 (30)	tab/cap	250 mg	250	Yes	No	Yes	NSCLC	Chemical Drugs 5.1
Lapatinib	6.80 (31)	tab/cap	250 mg	1,250	No	No	Yes	Breast Cancer	Chemical Drugs 5.1
Apatinib	22.20 (32)	tab/cap	250 mg	1,250	No	No	Yes	Gastrointestinal Neoplasms	Chemical Drugs 1
Crizotinib	7.70 (33)	tab/cap	250 mg	500	No	No	Yes	NSCLC	Chemical Drugs 5.1
Nilotinib	12 (34)	tab/cap	200 mg	600	No	Yes	Yes	Leukemia	Chemical Drugs 5.1
Sunitinib	5.60 (35)	tab/cap	12.5 mg	33	No	No	Yes	Gastrointestinal Neoplasms	Chemical Drugs 5.1
Icotinib	10 (36)	tab/cap	125 mg	125	No	No	Yes	NSCLC	Chemical Drugs 1
Erlotinib	9.70 (37)	tab/cap	150 mg	150	No	Yes	Yes	NSCLC	Chemical Drugs 5.1
Axitinib	20.10 (38)	tab/cap	5 mg	10	No	No	Yes	Renal Cell Carcinoma	Chemical Drugs 5.1
Sorafenib	19.20 (38)	tab/cap	200 mg	800	No	No	Yes	Renal Cell Carcinoma	Chemical Drugs 5.1
Bortezomib	14 (39)	inj	3.5 mg	0.42	No	Yes	Yes	Multiple Myeloma	Chemical Drugs 5.1
Everolimus	4.90 (40)	tab/cap	5 mg	10	No	Yes	Yes	Renal Cell Carcinoma	Chemical Drugs 5.1
Chidamide	8.50 (41)	tab/cap	5 mg	9	No	No	No	Lymphoma	Chemical Drugs 1
Recombinant Human Endostatin	8.20 (42)	inj	15 mg	9	No	No	No	NSCLC	Chemical Drugs 1

mPFS, median progression-free survival; biological products 3.1, the application for marketing in China of biological products produced abroad which are listed abroad but not listed in China; biological products 1, therapeutic biological products which are not listed in China or abroad; chemical drugs 5.1, the application for marketing in China of original brand drugs and improved drugs which should have obvious clinical-advantages; chemical products 1, the innovative drugs which are not listed in China or abroad.

### 2.3 Data collection

Trained research assistants systematically collected data on drug availability and pricing from medical institutions and retail pharmacies within the study area, utilizing a standardized form that captured essential information, including drug name, specifications, dosage form, packaging, and retail price during the survey.

### 2.4 Data analysis

#### 2.4.1 Availability assessment

According to the WHO/HAI definition, the availability of medicines was calculated as the proportion of all the surveyed institutions where a specific drug can be found (43).


$$\begin{array}{l} \text{Availability}\\ =\frac{\text{the number of institutions that}\;\mathrm{can}\;\text{provide the drug}}{\text{the total number of research institutions}}\times 100\% \end{array}$$

The following criteria were used to evaluate the availability of anticancer medicines [7]. 0% indicates that it is not available in any hospital surveyed. 0–30% means a very low availability. 30–49% indicate low availability. 50–80% had relatively high availability. 80–100% indicates high availability. In addition, the Wilcoxon rank-sum test method was used to analyze the difference in average availability in each region.

#### 2.4.2 Price evaluation

According to the WHO/HAI methodology, EAMs prices are evaluated by median price ratio (MPR). MPR is calculated using the following formula.


$$\mathrm{MPR}=\frac{\text{median price of drugs}}{\text{International reference price}}$$

The International Drug Price Guide of Management Sciences for Health is the international reference price (10). However, since reference prices for IAMs were unavailable in this guide, we utilized the median price derived from a cross-regional comparative study of IAM pricing in Europe, Australia, and New Zealand as an alternative benchmark (11). In addition, all drug specifications were normalized and compared based on minimum unit dosing (e.g., per milligram or international unit) to ensure consistent price evaluation. If MPR is between one and two, the price is acceptable; if MPR > 2, the price is high (12).

#### 2.4.3 Affordability assessment

We evaluated the affordability of anticancer medicines following the WHO/HAI methodological recommendations. The affordability of medicines is calculated by the wage of the lowest-paid unskilled government worker (LPGW). Considering the significant differences between urban and rural areas in China and the lack of data on LPGW, a modified WHO/HAI standard survey method was used. Therefore, we adopted the per capita disposable income in different cities, visited regional statistical bureaus’ websites, and compared each regions per capita disposable daily income in 2021. Cancer patients are classified under chronic disease management, with their affordability calculated as the ratio of the cost for a 30-day standard treatment course to per capita daily disposable income. For statistical analysis purposes, all dosage regimens are standardized according to the drug instructions. Considering local health insurance regulations and reimbursement policies, the national essential medical insurance program set the out-of-pocket payment ratio at 15% for urban employed individuals and 30% for rural individuals, respectively (13). Health expenditures were deemed affordable if they did not exceed Jiangsu’s per capita disposable income (daily wage). Conversely, if expenditures surpassed this threshold, they were considered unaffordable. The formula used to determine affordability is as follows:


$$\begin{array}{l} \text{Affordability}\\ =\frac{\text{total cost of}\;30\;\text{days of drug}\;\mathrm{use}\times \text{the ratio of out}-\text{of}-\text{pocket}}{\mathrm{per}\;\text{capita disposable income}(\text{daily wage})} \end{array}$$

A higher value indicates lower affordability, whereas a lower value suggests higher affordability. In this study, a value greater than 1 is deemed unaffordable. Conversely, if the value is less than or equal to 1, it is considered relatively good affordability.

For the convenience of calculation, the affordability evaluation criteria of IAMs are different from those of EAMs. We calculated the ratio of annual IAMs’ out-of-pocket expenses to catastrophic health expenditure. If the value is greater than 1, it is considered unaffordable. If the value is less than or equal to 1, it is considered relatively good affordability. In this study, we evaluated the duration of treatment with innovative anticancer medicines, IAMs per year, according to the median progression-free survival (mPFS, Table 2). If mPFS ≧ 1, we set the treatment duration to 1 year. If mPFS < 1, then mPFS represents the actual treatment duration per year. The IAMs for which mPFS cannot be obtained, the treatment duration was evaluated according to the treatment guidelines or relevant clinical trials. Catastrophic health expenditure means that the out-of-pocket medical expenses of families exceed the defined threshold of non-survival expenditure. It is considered that catastrophic health expenditure has occurred, and the threshold is set at 40% according to the WHO. Catastrophic health expenditure is important in measuring health equity (14).

#### 2.4.4 Comprehensive availability and affordability

A four-quadrant diagram was used to simultaneously display the results of the availability and affordability of medicines to identify the comprehensive situation. The availability value of the medicines for patients in rural or urban areas was shown on the *Y*-axis, while the affordability value was depicted on the *X*-axis. In the scattered plot, the upper left quadrant (I) showed the medicines with high availability and high affordability, the upper right quadrant (II) showed the medicines with high availability and low affordability, the lower left quadrant (III) showed the medicines with low availability and high affordability, the lower right quadrant (IV) showed the medicines with low availability and low affordability.

Data were analyzed using SPSS (version 26.0; SPSS, Inc., Chicago, IL, United States) and MATLAB (version 2018; MathWorks, Natick, MA, United States).

## 3 Results

### 3.1 Availability

**Figure 1:**
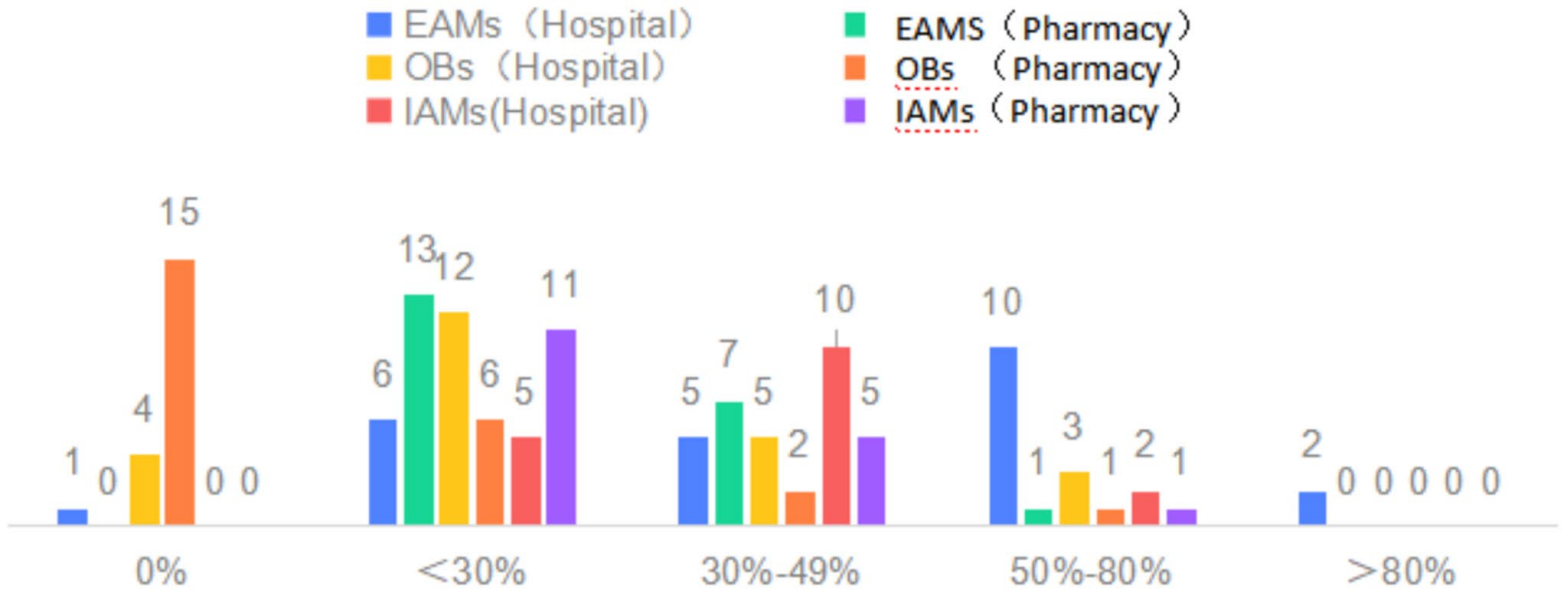
Availability distribution of EAMs and IAMs in hospitals and pharmacies (%).

Figure 1 showed the availability of LPGs, OBs and IAMs in hospitals and community pharmacies. In general, the availability of EAMs and IAMs was low, the average availability was basically below 50%. Among anticancer medications with high availability exceeding 80%, all were LPGs. For those with relatively availability between 50 and 80%, the majority were also LPGs. Importantly, for those anticancer medications showing availability above 50%, all the availability of LPGs, OBs and IAMs in hospitals were higher than that in community pharmacies.

The availability of anticancer medicines in high-income areas was significantly higher than in low-income areas. However, there was no statistical difference in the availability of IAMs in community pharmacies (*p* > 0.05). The average availability of LPGs in hospitals in different economic regions, which was significantly higher than that in community pharmacies, ranged from 41.25 to 59.31%, and even the availability of 5-FU and Oxaliplatin was over 80%. The average availability of OBs in hospitals in different economic regions, which was also higher than that in community pharmacies, ranged from 35.83–57.50% (Table 3).

**Table 3:** Availability of 41 anticancer medicines in nine cities.

Drug type	High-income areas (*n* = 20)	Middle-income areas (*n* = 15)	Low-income areas (*n* = 10)	*p*-value
EAMs (hospital)	51.67	59.31	41.25	0.001
EAMs (pharmacy)	24.38	19.44	17.08	0.008
OBs (hospital)	57.50	42.50	35.83	0.001
IAMs (hospital)	40.29	26.86	20.59	0.000
OBs (pharmacy)	24.17	15.00	5.00	0.000
IAMs (pharmacy)	29.71	27.84	27.06	0.454

In the community pharmacies, the availability of LPGs ranged from 0 to 64.44%, and among them, the drug with the highest availability was the oral targeted drug “Imatinib” (64.44%). There were three intravenous preparations with availability of less than 10%. We cannot get Etoposide, Carboplatin, and Mesna in pharmacies. The OBs in community pharmacies had the lowest availability, and we cannot get CTX, Cytarabine, and Mesna in pharmacies. Imatinib (53.33%), Trastuzumab (31.11%) and Rituximab (28.89%) had higher availability (Table 4).

**Table 4:** Availability of EAMs in hospitals and community pharmacies.

Drug name	LPGs		OBs	
	Hospitals	Pharmacies	Hospitals	Pharmacies
Cyclophosphamide	40.00	4.44	71.11	0.00
Ifosfamide	33.33	4.44	28.89	2.22
Methotrexate	57.78	40.00	N/A	N/A
Mercaptopurine	4.44	20.00	N/A	N/A
Cytarabine	35.56	4.44	37.78	0.00
Hydroxyurea	75.56	37.78	N/A	N/A
Fluorouracil	93.33	31.11	N/A	N/A
Gemcitabine	73.33	22.22	37.78	13.33
Etoposide	80.00	0.00	N/A	N/A
Daunorubicin	28.89	24.44	N/A	N/A
Vincristine	35.56	22.22	N/A	N/A
Paclitaxel	65.56	37.78	31.11	13.33
Cisplatin	15.56	20.00	N/A	N/A
Oxaliplatin	84.44	8.89	42.22	20.00
Carboplatin	11.11	0.00	22.22	2.22
Arsenic trioxide	4.44	4.44	N/A	N/A
Asparaginase	4.44	20.00	N/A	N/A
Calcium folinate	75.56	2.22	N/A	N/A
Capecitabine	75.56	48.89	53.33	37.78
Tamoxifen	80.00	37.78	N/A	N/A
Sodium mesylate	75.56	0.00	2.22	0.00
Imatinib	55.56	64.44	28.89	53.33
Rituximab	48.89	48.89	46.67	28.89
Trastuzumab	0.00	4.44	55.56	31.11
All drugs	48.10	21.20**	38.15	16.85**

N/A: drugs were not available which were not listed or had been withdrawn in China. **p < 0.01.

There was no statistical difference between IAMs in hospitals and community pharmacies (*p* > 0.05) (Table 5).

**Table 5:** Availability of IAMs in hospitals and community pharmacies.

Drug name	Hospitals	Pharmacies
Bevacizumab	33.33	31.11
Nimotuzumab	55.56	40.00
Dasatinib	6.67	20.00
Gefitinib	35.56	6.67
Lapatinib	2.22	20.00
Apatinib	31.11	66.67
Crizotinib	31.11	26.67
Nilotinib	13.33	31.11
Sunitinib	35.56	26.67
Icotinib	64.44	42.22
Erlotinib	33.33	13.33
Axitinib	37.78	24.44
Sorafenib	42.22	44.44
Bortezomib	32.22	20.00
Everolimus	22.22	20.00
Cedaniline	33.33	22.22
Recombinant human vascular endothelial inhibitor	24.44	28.89
All drugs (*p* = 0.327)	31.44	28.50

### 3.2 Price

Table 6 shows the information on the median price ratio of EAMs. Except for Suzhou and Lianyungang, the MPR values of LPGs were less than 2, and the MPR values of OBs were more than 5. However, there was no statistical difference in the median price ratio among cities.

**Table 6:** Analysis of MPR of EAMs in 45 hospitals in Jiangsu Province.

Drug type	High income areas				Middle income areas			Low income areas		*P*-value	95%Cl
	Nan-jing	Su-zhou	Wuxi	Chang-zhou	Xu-zhou	Yan-cheng	Yang-zhou	Huai-an	Lian-yungang		
LPGs	1.98	2.06	1.86	1.98	1.51	1.53	1.53	1.73	2.06	0.699	[−0.2864, 0.5664]
OBs	5.25	5.21	5.27	5.24	5.24	5.25	5.25	5.25	5.25	0.151	[−0.0325, 0.0125]

Tables 7, 8 evaluate and analyze the median price of IAMs. Among the nine IAMs that can be compared, except for everolimus, the median prices of drugs in community pharmacies and medical institutions are similar. In general, except for bevacizumab and bortezomib, the price of IAMs is higher than the median value of 18 countries, including Europe.

**Table 7:** Analysis of MPR of IAMs in 45 hospitals in Jiangsu Province.

Drug name	Dosage form	Specifications	Reference price (Euro)	Median price (Euro)	MPR
Bevacizumab	inj	100 mg	48.98	1.87	0.04
Bortezomib	inj	3.50 mg	1142.30	491.11	0.43
Erlotinib	tab/cap	150 mg	2.30	10.07	4.38
Gefitinib	tab/cap	250 mg	2.44	19.85	8.14
Everolimus	tab/cap	5 mg	1.92	16.17	8.44
Sunitinib	tab/cap	12.50 mg	0.12	1.54	12.68
Nilotinib	tab/cap	200 mg	0.001	0.06	40.42
Sorafenib	tab/cap	200 mg	0.28	11.82	42.05
Apatinib	tab/cap	250 mg	0.23	14.30	61.58
Nimotuzumab	inj	50 mg	N/A	178.48	N/A
Dasatinib	tab/cap	50 mg	N/A	14.44	N/A
Lapatinib	tab/cap	250 mg	N/A	8.29	N/A
Crizotinib	tab/cap	250 mg	N/A	28.46	N/A
Icotinib	tab/cap	125 mg	N/A	7.97	N/A
Axitinib	tab/cap	5 mg	N/A	24.46	N/A
Chidamide	tab/cap	5 mg	N/A	42.66	N/A
Recombinant human vascular endothelial inhibitor	inj	15 mg	N/A	60.94	N/A

**Table 8:** Analysis of MPR of IAMs in 45 community pharmacies in Jiangsu Province.

Drug name	Dosage form	Specifications	Reference price (Euro)	Median price (Euro)	MPR
Bevacizumab	inj	100 mg	48.98	1.87	0.04
Bortezomib	inj	3.50 mg	1142.30	490.99	0.43
Everolimus	tab/cap	5 mg	1.92	3.23	1.69
Erlotinib	tab/cap	150 mg	2.30	10.07	4.38
Gefitinib	tab/cap	250 mg	2.44	19.85	8.14
Sunitinib	tab/cap	12.50 mg	0.12	1.54	12.68
Nilotinib	tab/cap	200 mg	0.001	0.06	40.42
Sorafenib	tab/cap	200 mg	0.28	11.82	42.05
Apatinib	tab/cap	250 mg	0.23	14.30	61.58
Nimotuzumab	inj	50 mg	N/A	178.48	N/A
Dasatinib	tab/cap	50 mg	N/A	15.55	N/A
Lapatinib	tab/cap	250 mg	N/A	8.29	N/A
Crizotinib	tab/cap	250 mg	N/A	28.46	N/A
Icotinib	tab/cap	125 mg	N/A	7.97	N/A
Axitinib	tab/cap	5 mg	N/A	24.46	N/A
Chidamide	tab/cap	5 mg	N/A	42.66	N/A
Recombinant human vascular endothelial inhibitor	inj	15 mg	N/A	60.94	N/A

### 3.3 Affordability

The affordability data of 24 EAMs are shown in Table 9. Due to the low price of EAMs and the relatively light economic burden of patients, the affordability index of urban residents, whether in hospitals or community pharmacies, is less than 1. However, the affordability of rural residents is not high; the affordability of medical institutions is 1.09–2.95, and antitumor drugs in community pharmacies for rural residents is 1.24–2.52. The affordability of the OBs is worse than that of LPGs, and the median affordability value for rural areas is 48.26. For example, the affordability value of trastuzumab for rural residents in Lianyungang is 338.14. There were significant differences in affordability in medical institutions and community pharmacies in different regions (*p* < 0.05).

**Table 9:** Median affordability of EAMs in Jiangsu Province.

Area	Drug type	Low income areas		Middle income areas			High income areas				*p*-value
		Huai’an	Lianyun-gang	Xuzhou	Yan- cheng	Yang-zhou	Chang-zhou	Nanjing	Suzhou	Wuxi	
Urban	LPGs (hospital)	0.69	0.66	0.51	0.61	0.53	0.38	0.29	0.28	0.36	0.000
	LPGs (pharmacy)	0.63	0.64	0.60	0.56	0.40	0.34	0.32	0.39	0.44	0.000
	OBs (hospital)	12.02	13.25	12.93	12.06	10.37	8.02	7.18	6.23	7.49	0.000
Rural	LPGs (hospital)	2.95	2.66	1.92	2.21	2.11	1.46	1.39	1.09	1.34	0.000
	LPGs (pharmacy)	2.52	2.37	2.06	1.88	1.49	1.24	1.46	1.44	1.58	0.000
	OBs (hospital)	48.26	49.42	44.58	40.55	38.61	29.47	32.30	23.08	26.66	0.000

Tables 10, 11 show the affordability evaluation of 17 IAMs in hospitals and community pharmacies in nine cities. The price difference of IAMs in community pharmacies and hospitals is slight. For rural residents, only two drugs will not cause catastrophic health expenditure, namely, icotinib and everolimus; for urban residents, only three varieties caused catastrophic health expenditure: sorafenib, bevacizumab, and bortezomib. There was a significant difference in affordability between urban and rural residents (*p* < 0.01).

**Table 10:** Median affordability of IAMs in hospitals.

Drug name	Urban		Rural	
	Annual expenses (RMB)	Affordability	Annual expenses (RMB)	Affordability
Icotinib	2980.58	0.08	5961.17	0.35
Lapatinib	10519.27	0.28	21038.54	1.23
Gefitinib	10875.53	0.29	21751.05	1.27
Axitinib	22666.50	0.61	45333.00	2.64
Recombinant human vascular endothelial inhibitor	13522.02	0.37	27044.04	1.58
Erlotinib	11788.04	0.32	23576.08	1.37
Everolimus	7473.18	0.20	14946.35	0.87
Chidamide	24162.48	0.65	48324.96	2.82
Apatinib	35514.86	0.96	71029.72	4.14
Nimotuzumab	20281.74	0.55	40563.48	2.37
Sunitinib	20462.05	0.55	40924.09	2.39
Dasatinib	9217.42	0.25	18434.84	1.08
Nilotinib	34213.88	0.93	68427.76	3.99
Sorafenib	50175.07	1.36	100350.13	5.85
Bevacizumab	69435.17	1.88	138870.34	8.10
Bortezomib	57916.70	1.57	115833.40	6.76
Crizotinib	27620.61	0.75	55241.21	3.22
Mean (SD)	25225.01 (18872.72)	0.68 (0.51)**	50450.01 (37745.43)	2.94 (2.20)

^**^P < 0.01 Comparison of the affordability between urban and rural areas in hospitals.

**Table 11:** Affordability of IAMs in community pharmacies.

Drug name	Urban		Rural	
	Annual expenses (RMB)	Affordability	Annual expenses (RMB)	Affordability
Icotinib	3429.26	0.09	6858.53	0.40
Lapatinib	10763.04	0.29	21526.08	1.26
Gefitinib	11207.18	0.30	22414.36	1.31
Axitinib	22950.00	0.62	45900.00	2.68
Recombinant human vascular endothelial inhibitor	14127.58	0.38	28255.15	1.65
Erlotinib	11788.04	0.32	23576.08	1.37
Everolimus	7737.39	0.21	15474.77	0.90
Chidamide	24752.35	0.67	49504.71	2.89
Apatinib	36439.60	0.99	72879.20	4.25
Nimotuzumab	20615.30	0.56	41230.60	2.40
Sunitinib	20945.48	0.57	41890.95	2.44
Dasatinib	9839.00	0.27	19678.00	1.15
Nilotinib	34939.00	0.95	69878.00	4.08
Sorafenib	50175.07	1.36	100350.13	5.85
Bevacizumab	69435.17	1.88	138870.34	8.10
Bortezomib	58325.48	1.58	116650.96	6.80
Crizotinib	28543.72	0.77	57087.45	3.33
Mean (SD)	25647.8 (18829.34)	0.69 (0.51)**	51295.6 (37658.68)	2.9 (2.19)

^**^P < 0.01 Comparison of the affordability between urban and rural areas in social pharmacies.

### 3.4 Comprehensive analysis of availability and affordability

**Figure 2:**
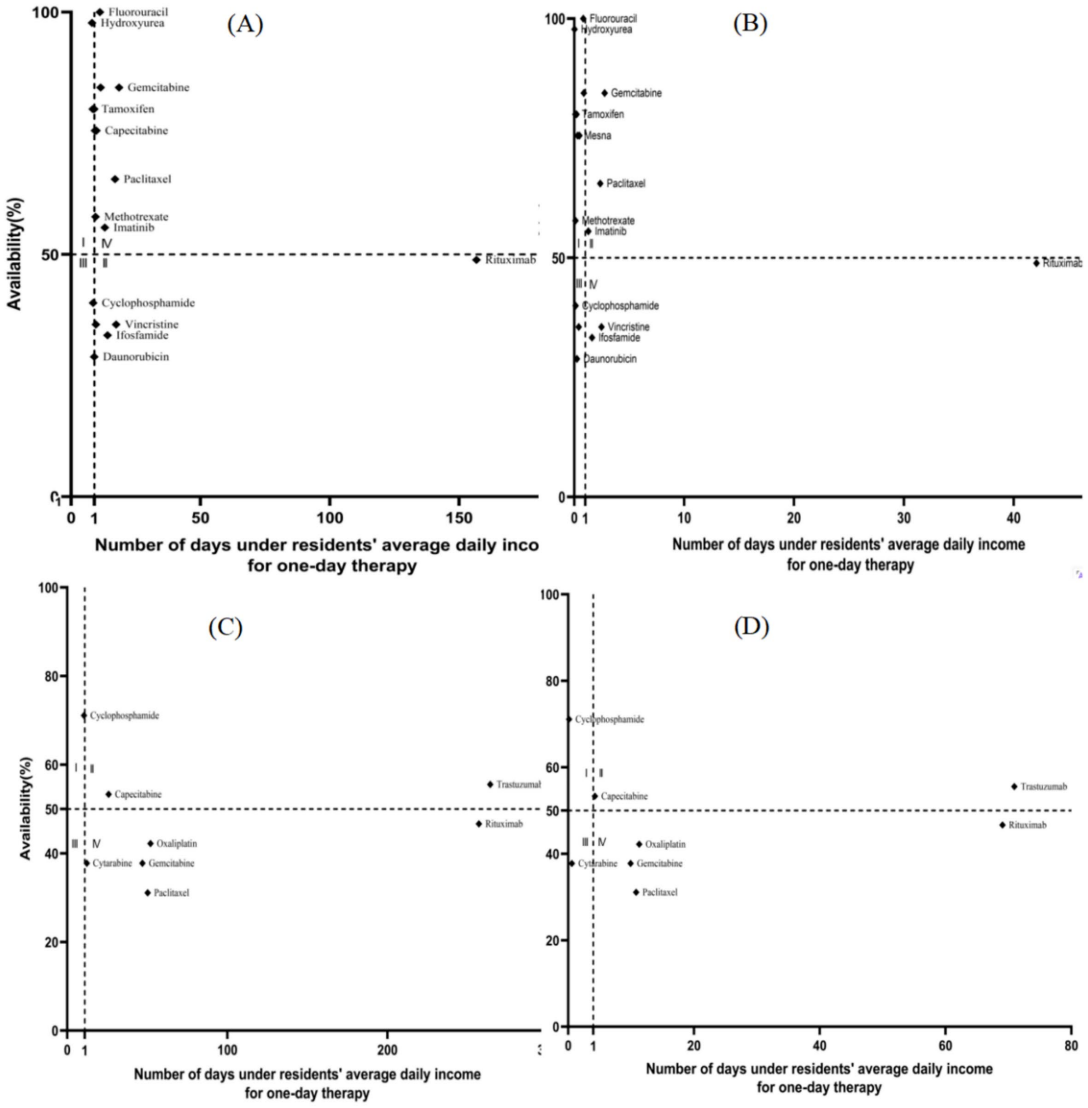
Comparison of the availability and affordability of EAMs (include LPGs and OBs) in hospitals. **(A)** Objective to analyze the availability and affordability of the LPGs in rural hospitals. **(B)** Objective to analyze the availability and affordability of the LPGs in urban hospitals. **(C)** Objective to analyze the availability and affordability of the OBs in rural hospitals. **(D)** Objective to analyze the availability and affordability of the OBs in urban hospitals.

**Figure 3:**
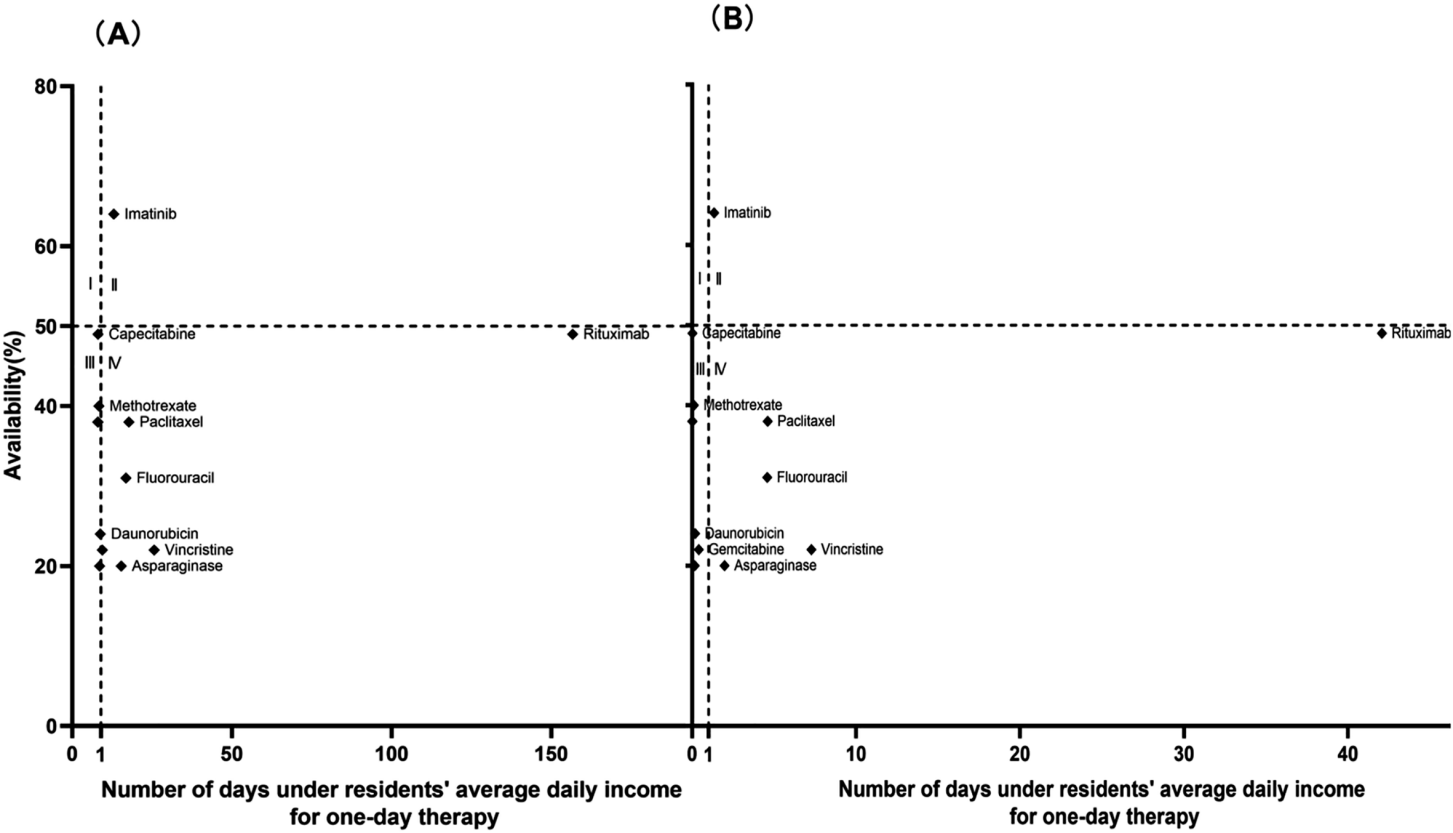
Comprehensive analysis of the availability and affordability of LPGs in community pharmacies. **(A)** Objective to analyze the availability and affordability of LPGs in rural community pharmacies. **(B)** Objective to analyze the availability and affordability of LPGs in urban community pharmacies.

**Figure 4:**
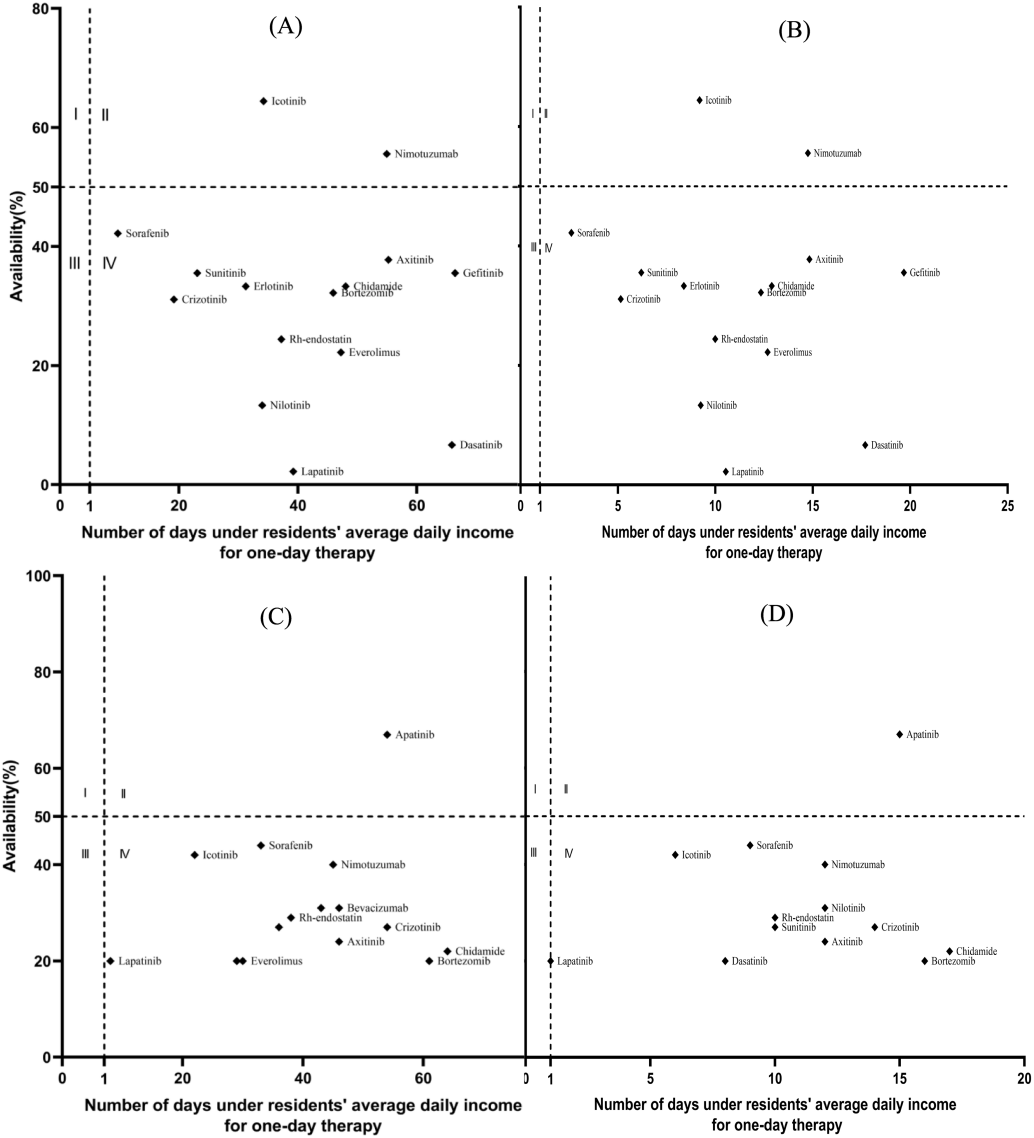
Comparison of the availability and affordability of IAMs. **(A)** Objective to analyze the availability and affordability of IAMs in rural hospitals. **(B)** Objective to analyze the availability and affordability of IAMs in urban hospitals. **(C)** Objective to analyze the availability and affordability of IAMs in rural community pharmacies. **(D)** Objective to analyze the availability and affordability of IAMs in urban community pharmacies.

Figures 2–4 present a four-quadrant diagram of the availability and affordability of different types of antitumor drugs in different scenarios. The number of varieties of basic drug generic drugs affordable to rural and urban residents is significant. However, the affordability of generic drugs in urban areas is better, and the affordability indicators of rural residents are mainly around the critical value. However, the affordability of the original research drugs of essential drugs for rural and urban residents is not ideal, and the availability is generally inferior to that of generic drugs. Only one new antitumor drug is available> 50% in community pharmacies, two in medical institutions, and only one variety with relatively good affordability in community pharmacies, lapatinib. In general, the burden of new antitumor drugs on rural residents is heavy, and the affordability of urban residents is relatively good. The availability of generic drugs for essential drugs in community pharmacies is generally lower than that in medical institutions. The availability of single varieties is roughly the same as in medical institutions, and community pharmacies are slightly higher.

## 4 Discussion

Using the WHO/HAI standardized survey methodology, this study evaluated the availability of anticancer medications across 45 healthcare facilities and 45 community pharmacies in nine municipal cities within Jiangsu Province, China. The main findings are summarized as follows.

First, the availability of anticancer drugs did not meet the targets set by the World Health Organization, with healthcare institutions serving as the primary channel for oncological treatment delivery. These results align with domestic studies examining the accessibility of essential anticancer medications in China (7). A cross-sectional study in Hubei Province reported a generic essential anticancer drug availability rate of 40.88%, compared to only 7.57% for originator products (6). The current study observed relatively higher availability rates for originator drugs, which can be attributed to the exclusion of 12 essential medications (e.g., methotrexate, tamoxifen, and hydroxyurea) whose originator formulations lacked regulatory approval in China—either never having been introduced or having been withdrawn from the market. Additionally, cross-sectional research involving 82 countries found that the median availability of the 20 most essential anticancer drugs in low- and middle-income countries (LMICs) ranged between 9 and 54% (15).

Although retail pharmacies serve as an important supplementary source of anticancer medications outside hospitals, healthcare institutions remain the primary cancer treatment providers, likely due to the specialized nature of oncology care. The study also identified significant regional disparities in drug availability across areas with differing levels of economic development. These variations may reflect inequalities in regional diagnostic and therapeutic capabilities (16), underscoring the need for policy interventions to address economic imbalances. Notably, no statistically significant regional differences were observed in the availability of novel anticancer agents in retail pharmacies, potentially due to the convenience and portability of oral dosage forms.

Second, the dosage form appears to be a critical factor influencing the availability of anticancer drugs in retail pharmacies. This study found significantly lower availability of essential medicines in retail pharmacies than in medical institutions. In contrast, novel anticancer drugs showed comparable availability between the two channels—a discrepancy that may be linked to differences in dosage forms. Among the 24 essential medicines assessed, 18 (75%) were injectable formulations, while only 4 out of 17 (23.5%) novel anticancer drugs were injectables. It is well established that intravenous infusions carry higher risks, with both long-established essential drugs and newly approved novel agents associated with infusion-related adverse reactions. According to 2020 national adverse drug reaction monitoring data, injections accounted for 56.7% of all reported adverse reactions (17). Furthermore, the July 2021 announcement by the Jiangsu Medical Insurance Bureau listed 100 dual-channel drugs, 37 of which were injectables (18), highlighting their importance in dual-channel recommended products. Due to the high-risk profile and complex clinical requirements of injectables, their availability through retail pharmacy supply chains remains limited. Therefore, improving the management system for “dual-channel” injectable drugs is crucial for enhancing the availability of injectable anticancer formulations. Pilot programs introducing day-care infusion centers have partially addressed this issue (19). For DTP (Direct-to-Patient) pharmacies, collaboration with medical institutions to establish day-care ward infusion centers represents a key strategy for overcoming infusion-related challenges in retail pharmacy supply models (20), thereby improving the availability of injectable formulations in community pharmacies.

Third, despite national reimbursement negotiations, the pricing and affordability of novel anticancer drugs continue to pose significant challenges. Between 2016 and 2020, 63 anticancer drugs were included in the national negotiated drug catalog, including 57 Western medicines with an average price reduction exceeding 44% (21). Among the 17 novel anticancer drugs assessed in this study, only lapatinib failed to renew its agreement in 2019; the remaining drugs were either included in the national negotiation catalog or directly incorporated into the standard reimbursement list. However, most novel anticancer drugs remain financially inaccessible to rural populations, necessitating further price adjustments aligned with China’s socioeconomic conditions to improve affordability.

Fourth, the suboptimal availability of negotiated drugs in medical institutions may be partially attributed to policy misalignment. Policy formulation should adequately consider stakeholder motivations and interests. This study’s relatively low availability of novel anticancer drugs appears closely associated with hospital performance evaluation metrics, including the assessment pressure on essential drug utilization rates. The introduction of novel anticancer drugs significantly impacts the quantity of essential medicines and financial proportion, particularly since most novel agents are high-cost drugs that substantially affect essential drug expenditure ratios. These pressures include public hospital performance evaluations, volume-based procurement targets, and drug expenditure control measures (22). Reimbursement policies also influence negotiated drug implementation. For example, using negotiated drugs may exceed Diagnosis-Related Group (DRG) payment limits, with hospitals bearing the financial overage. After incorporating novel anticancer drugs into reimbursement, further adjustments to insurance benefits should be made to reduce disparities between urban–rural resident insurance and urban employee insurance, improve cross-regional reimbursement policies, and provide appropriate support for cancer patients with high treatment costs but limited financial capacity. These measures are crucial for optimizing the inclusion of novel anticancer drugs in insurance coverage and implementing negotiated policies effectively (23). Additionally, reimbursement indication restrictions may play a role. As queried from the Jiangsu Medical Insurance Bureau website, the approved indications for negotiated drugs are strictly defined by insurance policies, creating higher utilization thresholds that further affect policy implementation. Policy coordination is equally critical for the efficient operation of DTP pharmacies (24). Performance evaluation targets across different departments require further alignment, particularly between healthcare insurance and health authorities. Establishing dedicated pathways for negotiated drug implementation and pre-publishing relevant evaluation standards could promote appropriate adoption and use in medical institutions. To address barriers related to evaluation metrics, incentive indicators such as negotiated drug adoption rates could be introduced. Additional measures could include excluding high-cost negotiated drugs from total insurance budgets, removing them from per-visit cost evaluations, and eliminating patient reimbursement caps to enhance policy coordination for negotiated drug implementation.

Fifth, the medication access model under the “Dual-Channel” policy remains inadequate. Jiangsu’s first Dual-Channel pharmacy catalog included only 225 designated pharmacies (25), compared to over 700 secondary and tertiary medical institutions (26). This limited pharmacy coverage affects patient access to negotiated drugs. Additional implementation barriers include external prescription processes, service and management capabilities, sales qualifications, and insurance coordination in retail pharmacies. As all negotiated anticancer drugs are prescription medications, the complex external prescription process creates significant inconvenience for patients and potential fraud risks, posing substantial regulatory challenges. Therefore, the government needs to optimize prescription access for patients purchasing medications at retail pharmacies, such as establishing dedicated third-party online consultation services for negotiated drugs where clinicians can conduct remote assessments and issue prescriptions directly to designated pharmacies. Alternatively, healthcare insurance and health authorities could designate qualified medical institutions to provide prescription services for patients requiring negotiated drugs, thereby improving coordination between inpatient and outpatient medication channels and streamlining purchasing processes. The study’s data collection period (July–November 2021) coincided with Jiangsu’s announcement of the Dual-Channel pharmacy catalog in August 2021, which may have contributed to the relatively low availability metrics observed.

## 5 Conclusion

The availability of anticancer drugs in Jiangsu remains suboptimal, with significant regional disparities observed across different economic zones, except for novel anticancer drugs in retail pharmacies. As an important supplement to medical institutions for medication supply, retail pharmacies generally demonstrate limited availability of anticancer drugs, particularly essential anticancer agents, likely due to their predominantly injectable formulations. For patients purchasing medications at DTP pharmacies, efficiently coordinating with medical institutions for intravenous administration presents a significant challenge. While national drug negotiations have substantially reduced prices for novel anticancer drugs, they remain financially burdensome for rural residents, necessitating further government-led price optimization to improve affordability. The dual-channel policy has partially alleviated pressure on negotiated drug adoption in medical institutions and increased market share. However, it requires further optimization in cross-departmental coordination and patient convenience.

This cross-sectional study conducted on-site investigations to evaluate anticancer drug availability across 45 medical institutions and 45 retail pharmacies in nine cities in Jiangsu Province. Compared to similar studies, this research innovatively included retail pharmacies as evaluation targets while assessing essential and novel anticancer drugs. The data collection period coincided with the initial implementation of Jiangsu’s Dual-Channel policy, potentially underestimating retail pharmacy availability. For most novel anticancer drugs lacking international reference prices, this study used median prices from 18 European countries; therefore, the calculated MPR values may not fully align with WHO/HAI standards. The affordability assessment only analyzed medication costs without including other treatment expenses, potentially overestimating patient capacity. For calculation purposes, the study assumed fixed reimbursement ratios (30% for rural and 15% for urban residents), which may not reflect actual reimbursement scenarios.

## Data Availability

The raw data supporting the conclusions of this article will be made available by the authors, without undue reservation.

## Glossary

DRG: Diagnosis-Related Group
DTP: Direct-to-Patient
EAMs: Essential Anticancer Medicines
EML: Essential Medicines List
GDP: Gross Domestic Product
GPCAIAMs: Guiding Principles of Clinical Application of Innovative Anticancer Medicines
LPG: Local procurement guideline
HAI: Health Action International
IAMs: Innovative Anticancer Medicines
IARC: International Agency for Research on Cancer
LMICs: Low- and Middle-Income Countries
LPGs: Lowest-priced Generics
LPGW: Lowest-Paid Government Worker
mPFS: Median Progression-Free Survival
MPR: Median Price Ratio
NEML: National Essential Medicines List
NHC: National Health Commission
NHSA: National Healthcare Security Administration
NRDL: National Reimbursement Drug List
OB: Procurement bid
OBs: Originator Brands
WHO: World Health Organization
